# Isolation and Characterization of Two Novel Genera of Jumbo Bacteriophages Infecting *Xanthomonas vesicatoria* Isolated from Agricultural Regions in Mexico

**DOI:** 10.3390/antibiotics13070651

**Published:** 2024-07-15

**Authors:** Claudia Villicaña, Lucía M. Rubí-Rangel, Luis Amarillas, Luis Alberto Lightbourn-Rojas, José Armando Carrillo-Fasio, Josefina León-Félix

**Affiliations:** 1 CONAHCYT—Laboratorio de Biología Molecular y Genómica Funcional, Centro de Investigación en Alimentación y Desarrollo, A. C., Culiacán 80110, Sinaloa, Mexico; maria.villicana@ciad.mx; 2 Laboratorio de Biología Molecular y Genómica Funcional, Centro de Investigación en Alimentación y Desarrollo, A. C., Culiacán 80110, Sinaloa, Mexico; lrubi221@estudiantes.ciad.mx; 3 Laboratorio de Genética, Instituto de Investigación Lightbourn, A. C., Cd. Jimenez 33981, Chihuahua, Mexico; l.amarillas@institutolightbourn.edu.mx (L.A.);; 4 Laboratorio de Nematología Agrícola, Centro de Investigación en Alimentación y Desarrollo, A. C., Culiacán 80110, Sinaloa, Mexico; acarrillo@ciad.mx

**Keywords:** bacteriophages, phage stability, bacterial spot, lytic, virulent, biocontrol

## Abstract

Bacterial spot is a serious disease caused by several species of *Xanthomonas* affecting pepper and tomato production worldwide. Since the strategies employed for disease management have been inefficient and pose a threat for environmental and human health, the development of alternative methods is gaining relevance. The aim of this study is to isolate and characterize lytic phages against *Xanthomonas* pathogens. Here, we isolate two jumbo phages, named XaC1 and XbC2, from water obtained from agricultural irrigation channels by the enrichment technique using *X. vesicatoria* as a host. We determined that both phages were specific for inducing the lysis of *X. vesicatoria* strains, but not of other xanthomonads. The XaC1 and XbC2 phages showed a myovirus morphology and were classified as jumbo phages due to their genomes being larger than 200 kb. Phylogenetic and comparative analysis suggests that XaC1 and XbC2 represent both different and novel genera of phages, where XaC1 possesses a low similarity to other phage genomes reported before. Finally, XaC1 and XbC2 exhibited thermal stability up to 45 °C and pH stability from 5 to 9. All these results indicate that the isolated phages are promising candidates for the development of formulations against bacterial spot, although further characterization is required.

## 1. Introduction

*Xanthomonas* is a Gram-negative bacterium, belonging to the Xanthomonadaceae family, which is widely distributed in all environments, including soils, water and plants. In the field of phytopathology, *Xanthomonas* species have emerged as important pathogens causing diverse plant diseases; bacterial spot is one of the most serious, affecting numerous crops in subtropical tropical regions and leading to important economic losses worldwide [1,2]. Recently, the causal agents of bacterial spot in tomato and pepper have been reclassified, being grouped into four lineages and three species: *X. euvesicatoria pv. euvesicatoria* (previously *X. euvesicatoria*), *X. euvesicatoria pv. perforans* (previously *X. perforans*), *X. vesicatoria* and *X. hortorum pv. gardneri* (previously *X. gardneri*) [3,4]. Several strategies have been employed for the management of bacterial spot, including the use of healthy seeds and transplants, seed treatment, crop rotation, culture practices, growing less susceptible cultivars and applying copper compounds or antibiotics [5,6,7,8]. However, most of these methods have been inefficient for controlling the disease; in addition, the overuse of antibiotics has accelerated the emergence of *Xanthomonas* pathovars that are resistant to several antibiotics such as streptomycin, kanamycin, oxytetracycline, ampicillin and penicillin [9,10], posing a challenge to disease management and raising public concern regarding the impact on environment and the potential effects on human health.

Bacteriophages (or phages, for short) are viruses that naturally infect and kill bacteria. They are globally distributed and can be virtually isolated from diverse samples where their host bacteria are present. Phages may have different life cycles, including the lytic cycle, in which the phage infects its host bacteria and hijacks their metabolism for the production of new viral particles triggering bacterial lysis. Alternatively, phages may integrate into the bacterial genome and replicate, together with bacteria harboring the lysogenic cycle [11]. Because of the potential of lysing bacteria, phages have gained interest as an environmentally friendly alternative strategy to control bacterial infections, including in agricultural approaches for improving the management of phytopathogens making more sustainable food production [12].

Specific phages infecting xanthomonads have been isolated and characterized, and some of them have been evaluated in biocontrol applications in several crops, such as peach, cabbage and tomato (Reviewed in [13]). Most of the described phages for bacterial spot possess lytic activity against *X. euvesicatoria* [14,15], but few phages are lytic against *X. perforans* [16,17]. So far, only four studies have described phages presenting lytic activity against *X. vesicatoria*, resulting in only five phage genomes being available in the GenBank database: XaF13 (MN335248), phiXaf18 (MN46129), vB_XveM_DIBBI (NC_017981, JN022534), Eir4 (OL581611) and Eisa9 (OL58161) [18,19,20]. Notwithstanding, a commercial formulation marketed as Agriphage^TM^ for bacterial spot in tomato and pepper has been developed by Omnilytics [2]. Hence, genetic information about specific *X. vesicatoria* phages has been underexplored, highlighting the lack of knowledge about their diversity and biology, and limiting the development of formulations for biocontrol applications.

Herein, we describe two novel genera of jumbo phages (XaC1 and XbC2) with lytic activity against *X. vesicatoria*, which were isolated from water obtained from irrigation channels in tomato fields in Sinaloa, Mexico. We report their morphology, genome sequences and annotation, phylogenetic and comparative analyses and stability to temperature and pH. All taken together, our results indicate that these phages present unique properties that make them promising agents for the biocontrol of pathogenic bacteria.

## 2. Results

### 2.1. Isolation and Morphology of X. vesicatoria Phages

**Figure 1:**
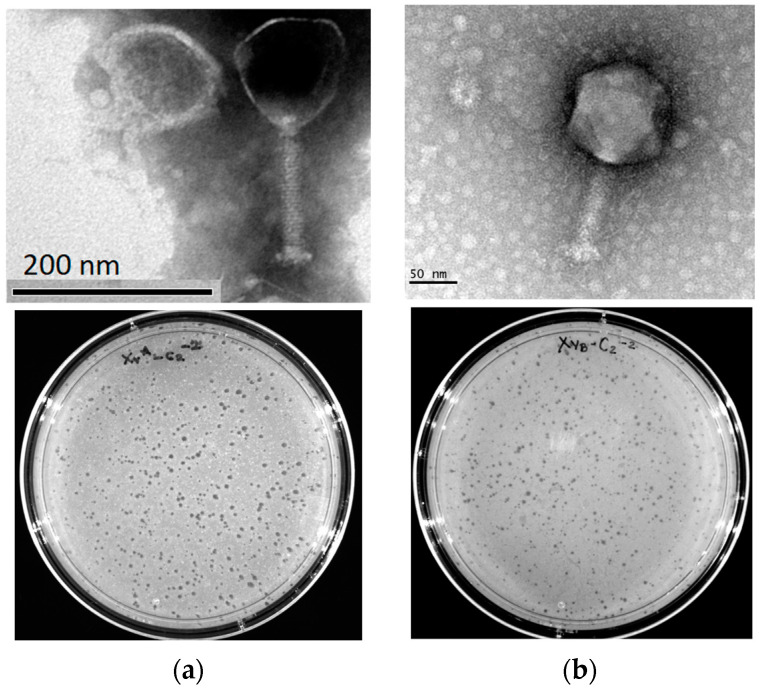
Morphology of *X. vesicatoria* jumbo phages (**a**) XaC1 and (**b**) XbC2. Lytic plaques were observed by double agar overlay done with TSB top agarose (0.4%) over TSA medium and incubated at 27 °C for 18 h.

Two phages were isolated after the enrichment of water, with *X. vesicatoria* strain A or B, obtained from irrigation channels; these were named XaC1 and XbC1. The Xa or Xb prefix denotes the strain used for enrichment; meanwhile, C1 or C2 indicates the irrigation channel from which the water sample was taken. Both the phages produced clear and defined plaques, indicative of lytic phages, showing small lytic plaques of about 1 mm for XaC2 and XbC2. Micrographs revealed that both phages were non-enveloped with icosahedral capsids, whereas XaC1 had a contractile tail. XaC1 exhibited a capsid of 110 nm and a 133 nm × 22 nm tail; XbC2 exhibited a head diameter of 125 nm and a 106 nm × 19 nm tail (Figure 1). According to these features, both phages exhibited a myovirus morphology.

### 2.2. Host Range in X. vesicatoria, X. euvesicatoria pv. euvesivatoria and X. hortorum pv. garneri

The host range of the two phages was evaluated with 28 strains, belonging only to three *Xanthomonas* species, including the 5 *X. vesicatoria*, 21 *X. euvesicatoria pv. euvesivatoria* and 2 *X. hortorum pv. garneri* strains. Our results showed that phage XaC1 was able to lyse three *X. vesicatoria* strains (XvA, XvB and Xv-17) but no strains belonging to *X. euvesicatoria* or *X. hortorum pv. gardneri*. Meanwhile, XbC2 lysed XvA, XvB and Xv-2 strains from *X. vesicatoria*, but no strains for other species (Table 1). These results indicate that the XaC1 and XbC2 phages exhibited narrow host ranges specific for the lysis of certain *X. vesicatoria* strains but not for other species of xanthomonads.

### 2.3. Restriction Analysis of Phage

**Figure 2:**
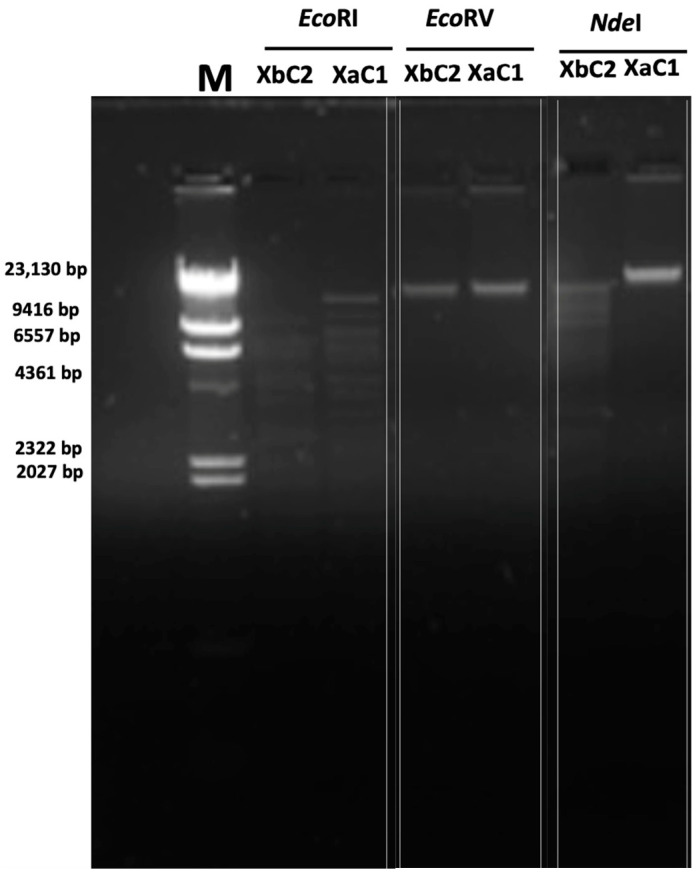
Restriction analysis of DNA from XaC1 and XbC2 phages. M, molecular weight marker.

The extracted DNA of XaC1 and XbC2 was analyzed prior to sequencing with *Eco*RI, *Eco*RV and *Nde*I to determine if specific digestion patterns could be found. The DNA from XaC1 showed several restriction fragments with *Eco*RI but was undigested with *Eco*RV and *Nde*I, whereas DNA from XbC2 showed restriction patterns when digested with *Eco*RI and *Nde*I (Figure 2). These findings strongly supported that each phage represents a unique entity and confirmed that the phage genomes are double-stranded DNA.

### 2.4. General Genome Features

Raw reads were filtered and assembled with SPAdes, resulting in a single contig for each phage, and showing higher percentages of mapping (about 98%) with Bowtie2 using the assembled genome as a reference (Table S1). Regarding termini, the XaC1 and XbC2 phages were analyzed with PhageTerm, revealing that both phages have redundant and non-permuted direct terminal repeats (DTR) and long ends type T5; the DTR were 32,031 bp and 17,581 bp in length in XaC1 and XbC2, respectively (Supplemental Materials S1 and S2). The assembled genomes of the two Xanthomonas phages showed genome sizes larger than 200 kb, indicating they represent jumbo phages (Table 2). A survey in the GenBank database revealed that XbC2 and XaC1 are the largest Xanthomonas phages reported, with 367,901 bp and 348,967 bp, respectively, even though the phage XacN1 has been reported with a genome size of 384,670 bp (revised October 2023) [21]. The XacN1 genome length includes 65,875 bp DTRs at the start and at the end of the reported genome; meanwhile, the XaC1 and XbC2 genome length was calculated considering only one DTR at the start of genome, as we mentioned in the Section 4.

**Figure 3:**
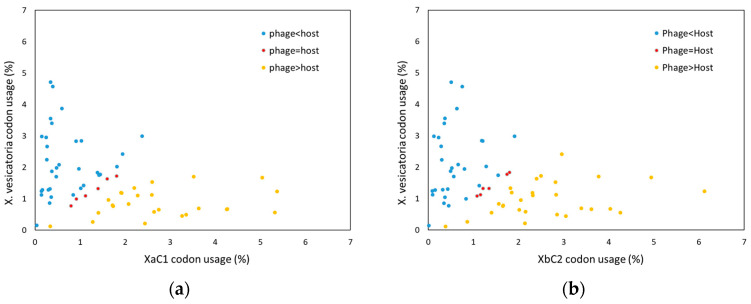
Comparison of codon usage between *Xanthomonas* phages and *X. vesicatoria*. (**a**) XaC1; (**b**) XbC2. Blue dots represent codons where the tRNAs have a higher codon usage in the host; in red, codons where the tRNAs has a similar usage in phage and host; in yellow, codons where the tRNAs showed a higher usage in the phage.

The G+C content of XaC1 and XbC2 was 35.1 y 37.5%, respectively, a lower value in comparison to that of representative Xanthomonas species, which is about 65% [22]. Phage XaC1 encodes 550 ORFs, of which 426 were annotated as hypothetical proteins. Meanwhile, XbC2 encodes 557 ORFs, and 416 were predicted as hypothetical proteins (Tables S2 and S3). The DTR regions were found to encode 70 ORFs for XaC1 and 35 ORFs for XbC2, all encoding hypothetical proteins. Interestingly, Xanthomonas phages contained several genes encoding for tRNAs. The XaC1 phage was found to contain eight genes encoding for tRNAS, whereas XbC2 contained 34 genes encoding for tRNAs, indicating that Xanthomonas phages encode their own tRNAs independently from the host (Table 1, Tables S4 and S5). Codon usage analysis revealed that XaC1 and XbC2 exhibited a higher usage of 25 and 28 tRNAs than the host, respectively; meanwhile, only six tRNAs showed a similar usage, and 33 and 30 tRNAs were more frequently used by the representative host (Figure 3). Moreover, five tRNAs encoded by XaC1 were classified within the higher codon usage phage group, while two tRNAs showed a similar codon usage and only one tRNA was found with a higher usage by the host. In the case of XbC2, 16 tRNAs were located in the higher codon usage phage group, six tRNAs showed a similar codon usage and 12 tRNAs encoded by XbC2 showed a higher codon usage by the host (Tables S6–S8).

The prediction of lifestyle with PHACTS suggested that XaC1 and XbC2 are lytic phages, consistent with the presence of clear plaques observed in double agar plates (Figure 1). Moreover, analyses in VFBD, ResFinder and Virulence Finder revealed that the XaC1 and XbC2 phages did not contain genes encoding potential virulence factors or antibiotic resistance genes. In silico restriction analysis of the phage genomes revealed that XaC1 and XbC2 have several restriction sites with the three enzymes used in this study (Supplemental Materials S3 and S4), which is inconsistent with the inability of EcoRV and NdeI to cut XaC1 and XbC2 genomes (Figure 2). These data may suggest that these viral genomes can be chemically modified, a feature common in DNA viruses, thus inhibiting endonuclease activity [23].

### 2.5. Detailed Analysis of Putative ORFs

#### 2.5.1. Structural Genes, DNA Packaging and Lysis

The XaC1 an XbC2 phages encode several structural proteins, including major head protein, tail, neck, tail sheath, head completion, long tail fibers and assembly proteins, baseplate, tail tube and other structural proteins. Moreover, some structural proteins showed enzymatic activity, such as tail lysozymes (ORF315 for XaC1; ORF236 and ORF237 for XbC2), prohead core scaffold proteases (ORF332) and head maturation protease (ORF515) for XaC1. Both phages encode one tail assembly chaperone and a cochaperonin type GroES, which have participated in promoting protein folding and morphogenesis. In the case of DNA packaging, both phages harbor two large terminase homologs that were found in tandem (ORF353 and ORF354 for XaC1; ORF270 and ORF271 for XbC2) and the portal protein (ORF248, XbC2; ORF329 XaC1), but no small terminase subunit was found. In addition to tail-associated lysozymes, phage XaC1 encodes for a rz-like spanin (ORF226) and an EPS depolymerase (ORF298) with a pectin_lyase fold (IPR011050), making the latter possibly important for biofilm disruption and polysaccharide degradation during adhesion [24]. Meanwhile, XbC2 encodes an O-spanin (ORF1159) and a lysozyme (ORF184) (Tables S9 and S10).

#### 2.5.2. DNA Metabolism, Replication, Repair and Recombination

The XaC1 and XbC2 phages encode several proteins involved in DNA replication, including DNA polymerases (XaC1, ORF342 and ORF411; XbC2, ORF259 and ORF276), which showed a high similarity to other phage polymerases. Moreover, the replication machinery of these Xanthomonas phages includes DNA ligases, ssDNA binding proteins, DNA primases, helicases, clamp loaders, topoisomerases and RNases. Meanwhile, for DNA metabolism, both phages encode for several genes involved in biosynthetic pathways, such as thymidylate synthase, dihydrofolate reductase and CMP/dCMP deaminase, and other enzymes such as cytidyltransferases and nucleotidyl transferases (Tables S9 and S10). In addition, several nudix and Mut/NUDIX hydrolases were identified in both phages; these proteins have been associated to nucleotide metabolism and DNA oxidative damage repair in bacteria [25]. Other repair genes are 2OG-Fe(II) oxygenase (ORF458 in XaC1), pyrimidine dimer DNA glycosylase/endonuclease V (ORF528 in XbC2) and phosphoglycolate phosphatase (ORF329 in XbC2), which may be involved in DNA activation repair pathways due to alkylation, UV-induce lesions and the formation of 2′phosphoglycolate [26]. In the case of recombination, XaC1 and XbC2 encode one RecA-like protein and recombination-related endonucleases, which can be important during replication and repair (Tables S9 and S10).

#### 2.5.3. DNA Cleavage and Modification

Several HNH endonucleases are encoded in XaC1 (ORF228 and OF288) and XbC2 (ORF381, ORF465 and ORF518), which have been suggested to participate in recombination and the DNA packaging process [27]. Moreover, XbC2 encodes other additional endonucleases (ORF322, ORF323 and ORF500) and exonucleases (ORF351 and ORF381). Regarding modifying enzymes, XaC1 phage contains a putative N-6-adenine-methyl transferase (ORF104) and a S-adenosyl-L- methionine(SAM)-dependent methyltransferase (ORF538), whereas XbC2 encodes a putative adenine methyltransferase (ORF56), a SAM-dependent methyltransferase (ORF125), a site-specific DNA methyltransferase (ORF148) and a DNA cytosine methyltransferase (ORF400). The presence of genes involved in DNA modification may mediate phage resistance against the restriction-modification bacterial system, providing protection against endonuclease activity [23,28] (Tables S9 and S10).

#### 2.5.4. Transcription, Translation and tRNA Processing

XaC1 and XbC2 phages encode some proteins related to transcription. XaC1 encodes two sigma factors (ORF320 and ORF541), whereas XbC2 encodes transcriptional regulators (ORF502 and sigma factors ORF241 and ORF504); meanwhile, for translation, XaC1 encodes a translation inhibitor (ORF256) and a translation initiation factor (ORF402) and XbC2 encodes the ribosomal protein S30EA (ORF185) and the translation factor IF-3 (ORF314). In addition to tRNAs, the XaC1 and XbC2 phages encode several proteins related to tRNA biosynthesis and function, such as the attachment of the corresponding amino acid onto its tRNA by tRNA synthetase/ligase, tRNA processing by tRNA nucleotidyl transferase/tRNA-His guanylyltransferase, the release of tRNA from a peptidyl-tRNA by peptidyl-tRNA hydrolases and the conversion of incorrectly acylated tRNAs by amidotransferase (Tables S9 and S10) [29].

#### 2.5.5. Miscellaneous Functions

In this category, we describe several genes that encode for specialized functions. In XbC2, ORF121 and ORF124 were associated to MazG toxin/antitoxin systems, as well as defense proteins associated to CRISPR/Cas system-associated protein Cas4 (ORF296) and Sir-2 (ORF109). Other proteins with hydrolase, ATPase and phosphatase activities were common in both phages. Regarding stress responses, XaC1 and XbC2 were found to encode PhoH-like and starvation-inducible DNA proteins which may mediate responses in low nutrient conditions [30]; ORFs encoding subunits of Clp proteases were found in both phages, which have been reported in prophage induction [31]. Moreover, XaC1 also encoded an anion resistant protein (ORF434) and a TerD family protein (ORF432), the latter may be related to tellurium resistance [32]; in addition, XbC2 encoded a toxic ion resistance protein (ORF336) and a metallopeptidase (ORF338) (Tables S9 and S10).

### 2.6. Phylogenetic and Comparative Analysis

**Figure 4:**
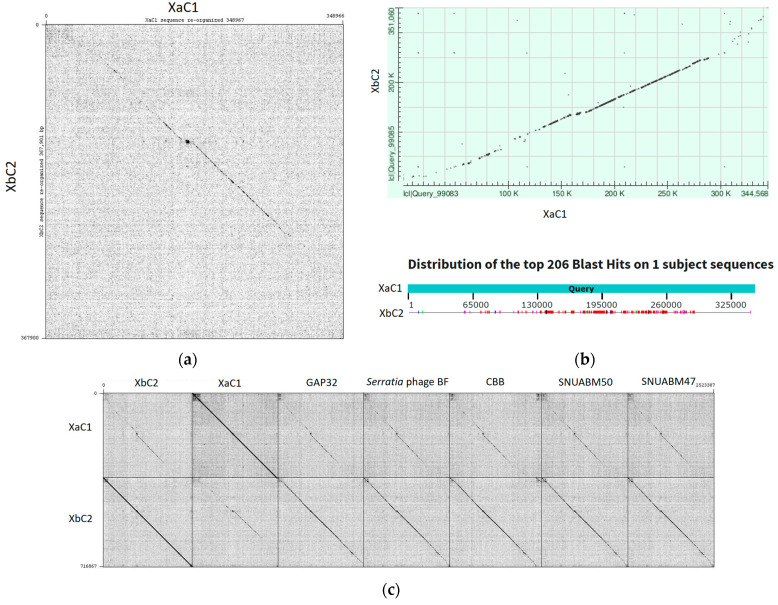
Dot plot analysis of XaC1 and XbC2 against similar phages previously identified by BLASTn analysis. Dot plot analysis of XaC1 vs. XbC2 visualized with (**a**) Gepard and (**b**) Blastn. (**c**) Multiple dot plots of XaC1 and XbC2 compared with other similar phages. GAP32, Cronobacter phage vB_CsaM_GAP32; Serratia phage BF; CBB, Pectobacterium phage vB_PcaM_CBB; SNUABM_50, Erwinia phage pEa_SNUABM_50; SNUABM_47, Erwinia phage pEa_SNUABM_47.

BLASTn analysis of complete genomes using Caudovirales taxid revealed that phage XaC1 had the best match with the Pectobacterium phage vB_PcaM_CBB (NC_041878.1, E-value 0.0), showing an identity of 74.50% but a low query cover of 5%. Meanwhile, XbC2 showed the best match to the Cronobacter phage vB_CsaM_GAP32 (JN882285.1, E-value 0.0), showing an identity of 78.90% and a high query cover of 47%. The comparison of the XaC1 and XbC2 genomes by dot plots using Gepard revealed that both phages were similar along the genome except at the genome ends (Figure 4a); this was further supported by pairwise alignment using BLASTn (with algorithm parameters of somewhat similar sequences), which showed a query cover of 30% and an identity of 66.91% with the two phages exhibiting similar dotplot patterns (Figure 4b). Intergenomic similarity was evaluated by Viral Intergenomic Distance Calculator (VIRIDIC), revealing that XaC1 and XbC2 shared only 21.2% similarity, indicating that both phages do not belong to the same genus. Moreover, XaC1 and XbC2 were compared against other similar phages infecting *Cronobacter*, *Serratia*, *Pectobacterium* and *Erwinia* obtained from previous BLASTn analysis, showing that XaC1 has an intergenomic similarity of about 21–22%, similar to the comparison with XbC2; meanwhile, XbC2 possesses a higher intergenomic similarity than the other analyzed phages, of above 50% (Table S11). Similar results were observed comparing the dot plots of XaC1 and XbC2 to those of phages from *Cronobacter*, *Serratia*, *Pectobacterium* and *Erwinia*, which showed that XaC1 was more dissimilar than XbC2 when compared with the additional phages (Figure 4c). 

**Figure 5:**
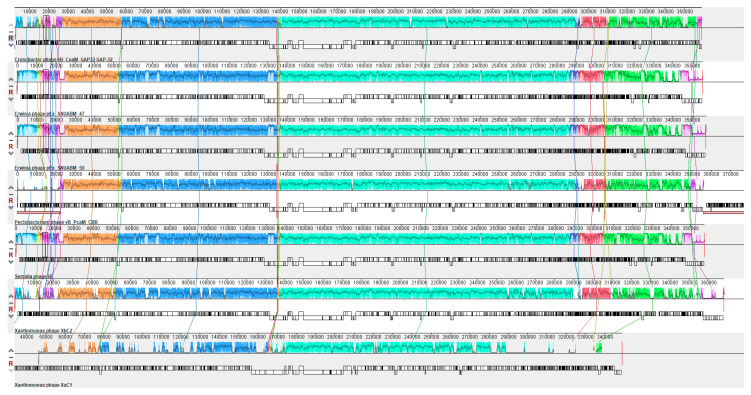
Mauve alignment analysis of XaC1 and XbC2 phages. From top to bottom: Cronobacter phage vB_CsaM_GAP32; Erwinia phage pEa_SNUABM_47; Erwinia phage pEa_SNUABM_50; Pectobacterium phage vB_PcaM_CBB; Serratia phage BF; Xanthomonas phage XbC2; Xanthomonas phage XaC1.

Mauve alignment analysis revealed that XaC1 and XbC2 phages showed a conserved modular structure, observed by shared collinear blocks throughout the genomes of the Cronobacter, Serratia, Pectobacterium and Erwinia phages, although XaC1 was more dissimilar than XbC2, resulting in the lack of some blocks (Figure 5). Collectively, these results clearly suggest that despite both XaC1 and XbC2 maintain relatively conserved and homolog modules, and they represent a novel genus of phages.

The classification of phages was analyzed using ViPTree. This analysis revealed that the XaC1 and XbC2 phages clustered in a clade containing the previously identified Pectobacterium CBB, Cronobacter GAP32 and Serratia BF phages, and additionally, the Yersinia phage fHe-Yen9-04, which belongs to the Mimasvirus and Enelasduvirus genera from the Zobellviridae family. However, XaC1 and XbC2 represent two novel phage genera based on the fact that their intergenomic similarity is below 70%, the threshold established by the International Committee on Taxonomy of Viruses (ICTV) for novel genus identification [33]. 

**Figure 6:**
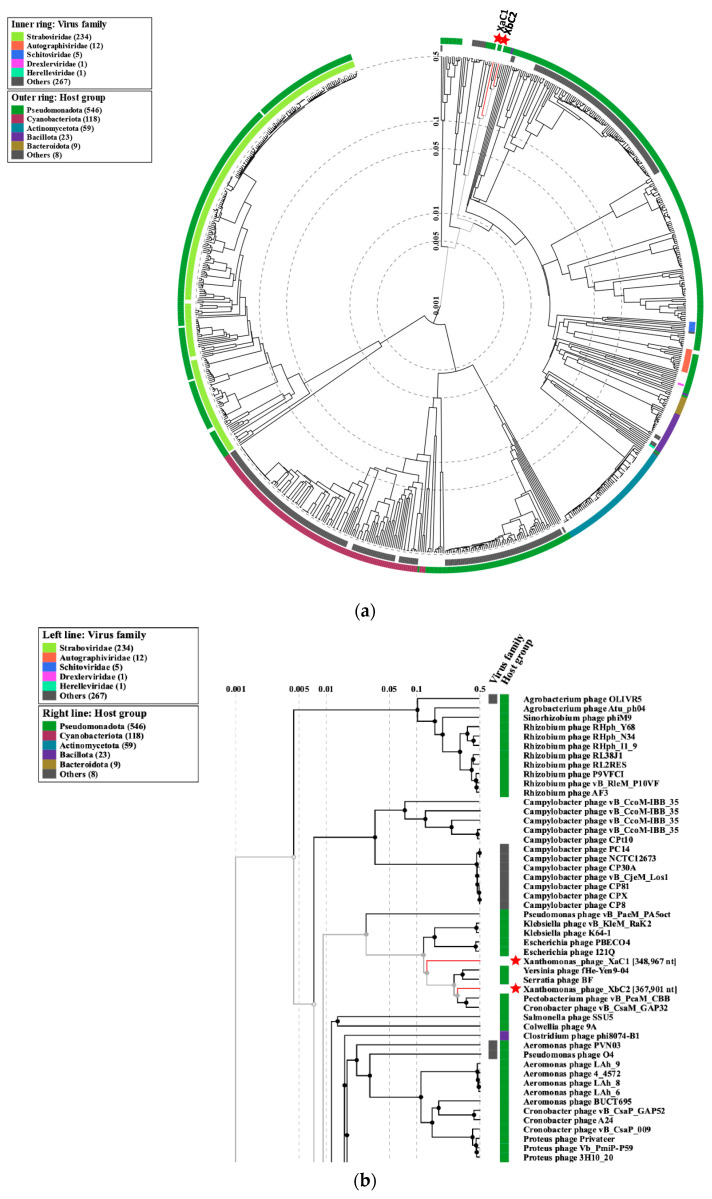
XaC1 and XbC2 phage proteomic tree using ViPTree in default mode. (**a**) Circular image of XaC1 and XbC2 phages. The red stars indicate the phage’s location. (**b**) The local zoom-in proteome tree structure of phages XaC1 and XbC2 reveals close viral relationships. The inner and outer colors indicate viral families and host groups, respectively.

**Figure 7:**
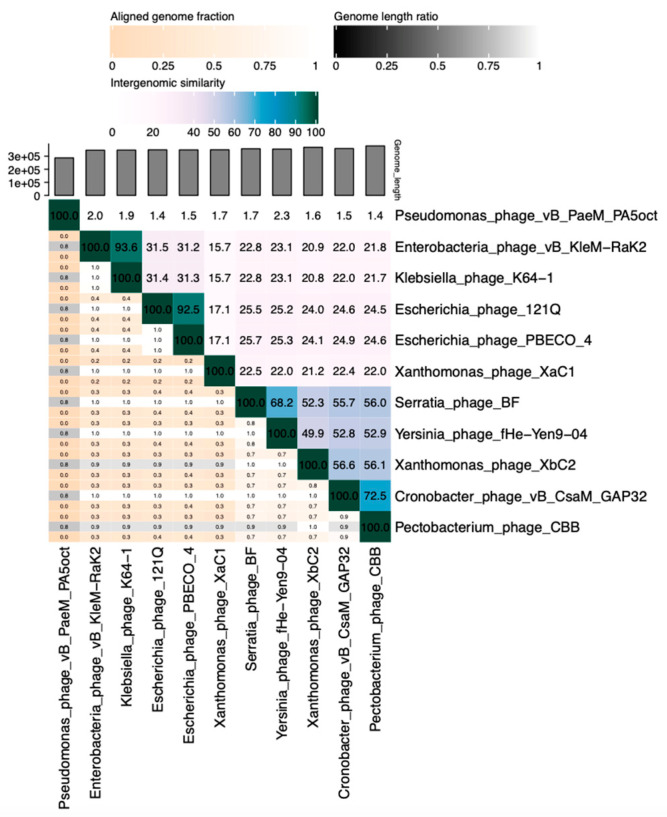
Intergenomic similarities between related phages of XaC1 and XbC2 generated by VIRIDIC. The right half of the image represents a visualization of phage genome clustering based on the similarity between intergenomic regions. Phages with darker colors are more closely related. The left half shows the three indication values of each pair of genomes. These values are presented in order: aligned partial genome 1 (row), genome length ratio (between genomes), and aligned partial genome 2 (column).

To determine if Xanthomonas phages belong to Zobellviridae family, we analyzed them with the member from the clade on ViPTree (Figure 6b) using VIRIDIC. As shown in Figure 7, the XbC2 phage belongs to the Zobellviridae family due to intergenomic similarity being higher than 50%, a value observed in members belonging to this family. In the case of XaC1, intergenomic similarity was very low, strongly suggesting it does not belong to Zobellviridae, and remains unclassified.

### 2.7. Thermal and pH Stability Assays

**Figure 8:**
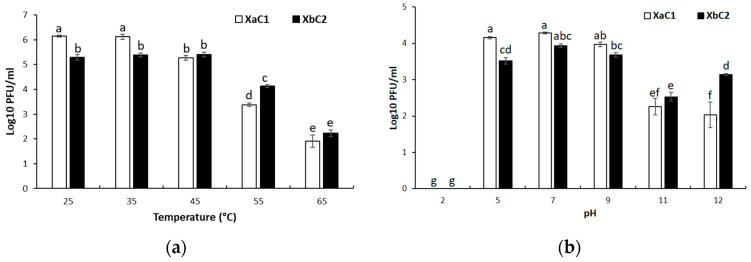
Stability assays for *Xanthomonas* phages: (**a**) Temperature, and (**b**) pH. Different letters indicate statistically significant differences (*p* < 0.05). Error bar represents standard deviation.

Factors such as temperature and pH largely influence phage viability, which may be a determinant for phage application. Thermal stability assays revealed that XaC1 showed no changes on infectivity between 25 °C and 35 °C but a significant reduction in titer of about <1-log unit at 45 °C (*p* > 0.05) compared with the initial titer (10^6^ PFU/mL); in contrast, phage XbC2 was stable at temperatures from 25 to 45 °C. At higher temperatures, XaC1 exhibited a reduction of 2.5-log units and XbC2 a reduction of 2-log units when exposed at 55 °C, showing XbC2 was significantly more stable than XaC1. However, both phages had a 4-log reduced viability at 65 °C (Figure 8a). 

Stability at different pH values showed that all phages lost viability at pH 2, resulting that no lytic plaques were obtained. The phage XaC1 was stable at the pH range from 5 to 9, but viability showed a significant reduction of >1-log at pH 11 and 12 after exposure for 24 h. The phage XbC2 was relatively stable at a pH range from 5 to 9, with less than a 1-log decrease in titer, similar to XaC1, but exhibited about a 2-log and 1-log reduction at pH of 11 and 12, respectively (Figure 8b). These findings clearly suggest that each phage displayed a different pH stability, and was more stable at a pH range from 5 to 9.

## 3. Discussion

Bacterial spot caused by *Xanthomonas* is a devastating disease, causing significant losses. Several studies have demonstrated that using lytic bacteriophages as treatments for controlling pathogenic bacteria is a promising strategy to overcome ineffective disease management and reduce the environmental impacts caused by chemical compounds [1]. Here, we isolated and characterized two novel jumbo phages specifically infecting *Xanthomonas vesicatoria* strains. The XaC1 and XbC2 phages have genome sizes of 348,967 nt and 367,901 bp, respectively, and were classified as jumbo phages due to possessing genomes larger than 200 kbp [34]. Both phages were lacking integrases or CI-like repressors, suggesting both phages are virulent, consistent with the formation of clear plaques onto top agar (Figure 1). Interestingly, the XaC1 and XbC2 phages possess long DTRs of 32,031 bp and 17,581 bp in length, respectively, suggesting the DTR packaging mechanism where the DTR is extended by synthesis of staggered nicks at the 3’ends [35]. Long DTRs have been found in several phages with lengths ranging from over hundred bases, such as the Tsamsa phage, at 284 bp [36], to several kilobases, such as the *Xanthomonas* phage XacN1, which shows a DTR length of 65,875 bp [21].

On the other hand, both phages were found to encode several tRNAs; in particular, XbC2 encodes 34 tRNAs, a feature shared with similar phages that we used for genomic comparisons, such as the *Pectobacterium* phage vB_PcaM_CBB (27 tRNAs), *Erwinia* phage pEa_SNUABM_50 (34 tRNAs), *Erwinia* phage pEa_SNUABM_47 (35 tRNAs), *Cronobacter* phage vB_CsaM_GAP32 (26 tRNAs) and *Serratia* phage BF (31 tRNAs). Generally, it has been proposed that phages encode tRNAs to compensate for codon usage, supplementing those codons essential for the phage but less commonly utilized by the host [37]. This can be supported by the fact that several XaC1 and XbC2 phage-encoded tRNAs showed a higher codon usage than the host (Tables S7 and S8). Moreover, other studies suggested that phage-encoded tRNAs can be positively selected because they are not sensitive to host anticodon nucleases [38] or used to evade retron-mediated bacterial immunity [39].

The genomic analysis of XaC1 and XbC2 revealed a relatively conserved modular arrangement compared with similar phages, identified by BLASTn and VIRIDIC, infecting *Erwinia, Serratia, Cronobacter* and *Cronobacterium*. Despite that a similar distribution of collinear blocks was observed using MAUVE alignment, the XaC1 phage showed a lower similarity to the other analyzed phages. Comparative genomics revealed that the XaC1 and XbC2 phages belong to different genera when they were compared with the best matches obtained by BLAST analysis, and, in fact, the XaC1 phage may represent a member of a novel family, broadening the diversity of phages known to date. Meanwhile, XbC2 is grouped as a member of the *Zobellviridae* family, where the additional phages analyzed for comparative genomics belonged to this same family. According to these results, we propose the two novel genera, “*Lururavirus*” and “*Villivirus*”, for the XaC1 and XbC2 phages described in this study, respectively.

On the other hand, the XaC1 and XbC2 phages contain a significant number of ORFs remains as ORFans encoding hypothetical proteins. Additionally, both phages harbor genes encoding methyltransferases, which may mediate phage protection against endonuclease activity of restriction-modification systems (Table S7) [40]. The phage XbC2 was found to encode Cas4 and Sir2-like proteins, which play roles in bacterial immunity. Cas4 is a DNA endonuclease implicated in the selection of a new spacer during CRISPR expansion [41]. Cas4 has been found in phages, including those that infect *Xanthomonas* species [40]. In *Campylobacter jejuni*, CRISPR systems lack Cas4 proteins. Still, phage-encoded Cas4 promotes the acquisition of new spacers of host origin, suggesting that acquiring self-spacers employs the bacterial genome as a decoy to prevent phage DNA acquisition [42]. Meanwhile, Sir2-like proteins have been reported in bacterial defense systems that deplete NAD^+^ and trigger abortive phage propagation [43].

The phage XaC1 encodes an EPS depolymerase (ORF298) harboring a pectin_lyase fold (IPR011050), a structure equivalent to ORF42 of phage SH-KP152226 from *Klebsiella pneumoniae* that encodes a depolymerase able to degrade exopolysaccharides (EPS) and biofilms [44], highlighting its potential as an enzybiotic. Other genes found in XaC1 and XbC2, such as TerD, phoH and MazG, have been reported as auxiliary metabolic genes (AMGs). TerD and phoH may contribute to increased host survival under harsh conditions, providing tellurite resistance and survival in low nutrient environments [45,46]. MazG is a pyrophosphohydrolase acting downstream of the MazEF toxin/antitoxin system, playing a role in decreasing cellular levels of the central alarmone (p)ppGpp in response to amino acid depletion [47]. MazG like proteins are commonly found in viral genomes from marine environments, and it is believed that depletion of cellular (p)ppGpp by viral MazG enables transcription in the infected cell, promoting viral replication as if it were in a nutrient-replete environment [48]. Recently, a study demonstrated that phage-encoded MazG-like proteins neutralize the host responses of TIR and STING, two abortive infection (Abi) systems, to ensure virus propagation [49].

Diverse environmental factors such as temperature and pH are known to inactivate phage particles due to structural protein damage. Considering that XaC1 and XbC2 can potentially be used for biocontrol, our results suggest that both phages remain relatively viable at a range of 25–45 °C and 5–9 pH. In Sinaloa, the temperature rarely exceeds 45 °C, and some studies reported that agricultural soils are moderately alkaline (pH 7.44), although acid and alkaline samples can be found in some locations [50,51]. Nonetheless, to overcome these limitations, phages can be formulated with polymers and other compounds to enhance their viability, ensuring effectiveness. Phage viability has been improved using trehalose, isoleucine, maltodextrin, chitosan and others [52,53,54]. Hence, the XaC1 and XbC2 phages are potential candidates for *Xanthomonas* biocontrol, so further studies must be done to reach their applications.

## 4. Materials and Methods

### 4.1. Bacterial Strains and Culture Conditions

*X. vesicatoria* strain A (XvA), *X. vesicatoria* strain B (XvB) and *Xanthomonas* strains used for host range were kindly provided by the Horticultural Laboratory at the Research Center for Food and Development Unit Culiacan (CIAD), A. C. (Table 1) [55]. All *Xanthomonas* strains were grown in tryptic soy agar (TSA) (BD Difco, Franklin Lakes, NJ, USA) plates or in trypticase soy broth (TSB) medium (Bioxon, Ciudad de Mexico, Mexico) at 27 °C under aerobic conditions. 

### 4.2. Isolation and Purification of Phages

Stems, leaves and soil from tomato plants presumptively infected with *Xanthomonas* showing symptoms of bacterial spot, as well as water samples from two irrigation channels (C1 and C2) were collected in tomato crops in the farm “Chaparral” located at Culiacan, Sinaloa, Mexico (24.603894, N-107.579580 coordinates) in June 2018. Stems, leaves and soil samples were mixed with sterile distillated water and shaken at 150 rpm at room temperature overnight. All samples were centrifuged at 8000× *g* for 15 min and the supernatant filtered through 0.22-μm sterile syringe filters of cellulose acetate membrane (GVS, USA). Phages were enriched as previously reported by Rombouts et al. [56] with some modifications. Briefly, 25 mL of overnight culture of XvA or XvB strains was supplemented with 5 mL of sample and incubated at 27 °C for 18 h. Then, 250 μL of chloroform were added and culture was incubated 1 h to lyse bacteria. Bacterial culture was centrifuged 5000× *g* for 30 min and the supernatant collected and filtered through 0.22 μm pore cellulose acetate membrane. Phages were detected by the double agar technique, in which 1 mL of XvA or XvB was added to 3 mL of molten TSB top agarose (0.4%) and mixed with 100 μL of enrichment filtrate, spread on TSA plates and incubated at 27 °C. Then, clear and non-turbid individual plaque was selected and recovered from the TSA plates with Pasteur pipettes. The plaque was resuspended in 1 mL of nanopure water and diluted to repeat the double agar for purifying the individual plaque. This step of purification was done at least eight times to obtain single-plaque isolates.

### 4.3. Propagation and Quantification of Phages

Phages were propagated according by the double agar overlay previously reported [57] with minor modifications. Briefly, the TSB top agarose containing the plaques of purified phage was recovered with sterile nanopure water and centrifuged at 8000× *g* for 15 min at 4 °C to eliminate the agarose. The supernatant was collected and centrifuged at 10,000× *g* for 15 min at 4 °C. Then, the resulting supernatant was recovered and centrifuged at 40,000× *g* for 2 h at 4 °C. The supernatant was discarded and the pellet resuspended in 10 mL of nanopure water and filtered in 0.22-μm sterile syringe filters of cellulose acetate membrane (GVS, Wisconsin, WI, USA). Phage quantification was done by serial dilutions and expressed as PFU/mL [58].

### 4.4. Transmission Electron Microscopy

Thirty microliters of purified phage (>10^8^ PFU/mL) were vacuum evaporated (JEE400, JEOL Ltd., Tokyo, Japan), negatively stained with 2% phosphotungstic acid (pH 7.2) and air dried. Phages were visualized on a JEOL JEM-1011 transmission electron microscope (JEOL Ltd., Tokyo, Japan) operated at 80 kV.

### 4.5. Host Range

Host range was determined by the drop method previously established [59]. Briefly, one milliliter of each *Xanthomonas* strains grown overnight was added to 3 mL of molten TSB top agarose (0.4% *w*/*v*), spread on TSA plates and place at rest until solidify. Then, a 10-μL drop of different phage concentrations (10^2^ to 10^8^ PFU/mL) was spotted onto the surface of plates and incubated overnight at 27 °C. The presence of a lysis zone and lytic plaques was considered as evidence of bacterial susceptibility to phages.

### 4.6. DNA Extraction and Restriction Analysis

Phage DNA extractions were done as previously described [60]. Briefly, one milliliter (10^8^ PFU/mL) was treated with DNAse and RNAse (Sigma-Aldrich, St. Louis, MO, USA) to remove exogenous DNA and RNA, then treated with SDS/proteinase K to remove proteins, and subsequently, DNA was extracted with phenol/chloroform extractions. For restriction endonuclease analysis, one microgram of DNA was digested with EcoRI, EcoRV, and NdeI according to the manufacturer’s instructions (Promega, Madison, WI, USA). Digested DNA was analyzed on 1% agarose gel in TAE, stained with ethidium bromide and documented with a ChemiDoC XRS imaging system (BioRad, Hercules, CA, USA).

### 4.7. Genome Sequencing, Assembly and Annotation

The DNA of phages (3 μg of each one) was sequenced using TruSeq DNA Nano protocol on an Illumina HiSeq platform, using 150 bp pair-end sequencing reads, performed at Laboratory of Genomic Services (LABSERGEN) in LANGEBIO-CINVESTAV (Irapuato, Mexico). Raw sequence reads were checked for quality using FastQC in the Galaxy platform (https://usegalaxy.org/, accessed on 30 January 2021) and trimmed with Cutadapt 1.16 (Parameters: Minimum length 20; Quality cutoff 30, 30; Trimm N’s on end of reads). Quality trimmed reads were de novo assembled into a linear contig using SPAdes 3.12.0 [61] with careful correction using different K-mer values (22, 33, 55, 77). The quality of assembly was evaluated using QUAST v5.0.2. and quality trimmed reads were mapped into the assembled genome using Bowtie2. Termini were determined with PhageTerm v1.0.12 [36] implemented in Galaxy from Pasteur Institute Platform (https://galaxy.pasteur.fr/, accessed on 23 February 2021) and genomes were reoriented placing DTRs only at the start of genome. Potential ORFs were predicted using GeneMarkS [62]. Functional annotation was done using BLASTP and PSI-BLAST algorithms at NCBI (minimum E-value: 1 × 10^−5^). Potential genes encoding tRNAs were predicted using tRNAscan-SE (ΔG~10 kcal/mol) [63] and ARAGORN [64]. Codon usage (r_i_) was calculated as previously reported by [21], using the *Xanthomonas vesicatoria* ATCC 35937 reference genome (Accession no. CP018725) as a representative genome of the host. Codon counts were calculated on the Codon Usage Calculator website (https://jamiemcgowan.ie/bioinf/codon_usage.html, accessed on 10 December 2023). Predicted proteins were also analyzed to identify possible virulence factors using the Virulence Factors Database (VFDB, http://www.mgc.ac.cn/VFs/, accessed on 30 April 2021) using a E-value cutoff of 1 × 10^−5^; and VirulenceFinder 2.0 (https://cge.cbs.dtu.dk/services/VirulenceFinder/, accessed on 24 March 2021); for antimicrobial resistance genes, we used ResFinder 4.1 (https://cge.cbs.dtu.dk/services/ResFinder/, accessed on 24 March 2021). The phage lifestyle was predicted with PHACTS (https://edwards.sdsu.edu/PHACTS, accessed on 3 March 2021) [65]. The genomes of phages XaC1 and XbC2 were deposited in GenBank under the accession numbers OR258281 and OP292654, respectively.

### 4.8. Phylogenetic and Comparative Genomics

Dot blot analysis was done to identify similarity or dissimilarity patters using Gepard with default parameters [66]. Comparative genomics of *Xanthomonas* phages was conducted with progressive alignment to determine conserved regions with MAUVE version 20150226 [67] and VIRIDIC [68] to determine genome similarity, in both cases using similar phage genomes such as *Pectobacterium* phage vB_PcaM_CBB (accession KU574722), *Erwinia* phage pEa_SNUABM_50 (accession MT939488), *Erwinia* phage pEa_SNUABM_47 (accession MT939487), *Cronobacter* phage vB_CsaM_GAP32 (accession JN882285) and *Serratia* phage BF (accession NC_041917). A comparative proteomic phylogenetic analysis was performed using a Viral Proteomic tree server [69] to compare phages from the Virus-Host database and NCBI GenBank database. 

### 4.9. Thermal and pH Stability Assays

To determine the thermal stability of phages, one milliliter of each phage (10^6^ PFU/mL) in SM buffer (50 mM Tris-HCl, pH 7.5; 8 mM MgSO_4_∙7H_2_O; 100 mM NaCl) was incubated at 25 °C, 35 °C, 45 °C, 55 °C and 65 °C, for 10 min, and then, the phage suspension was cooled at 4 °C and phage viability was determined by double agar overlay of decimal serial dilutions [68]. In addition, phage viability was investigated at different pH conditions, in which 10^4^ PFU phage particles were resuspended in 1 mL of SM buffer previously adjusted to pH 2, 5, 7, 9, 11 and 12, and incubated at 27 °C for 24 h. After incubation, phage viability was estimated by double agar overlay [70] and PFU/mL values were transformed to log10 PFU/mL. Thermal and pH stability assays were done in triplicate. Statistical analysis was carried out using two-way ANOVA (general linear model) with Bonferroni post hoc test in Minitab 17 software. Statistical differences were considered for *p* < 0.05.

## Figures and Tables

**Table 1:** Host range of jumbo phages infecting *Xanthomonas*. +, indicate lysis; −, indicate no lysis

Strains	Crop ^1^	XaC1	XbC2
*X. vesicatoria*			
XvA	Tomato	+	+
XvB	Tomato	+	+
Xv-2	Tomato	−	+
Xv-17	Tomato	+	−
Xv-44	Tomato	−	−
*X. euvesicatoria pv. euvesicatoria* ^a^			
Xe-1	Tomato	−	−
Xe-4	Tomato	−	−
Xe-5	Tomato	−	−
Xe-6	Tomato	−	−
Xe-7	Tomato	−	−
Xe-8	Tomato	−	−
Xe-11	Tomato	−	−
Xe-13	Tomato	−	−
Xe-15	Tomato	−	−
Xe-16	Tomato	−	−
Xe-18	Tomato	−	−
Xe-19	Tomato	−	−
Xe-24	Tomato	−	−
Xe-25	Tomato	−	−
Xe-26	Tomato	−	−
Xe-27	Bell pepper	−	−
Xe-30	Tomato	−	−
Xe-32	Bell pepper	−	−
Xe-34	Bell pepper	−	−
Xe-42	Tomato	−	−
Xe-46	Tomato	−	−
*X. hortorum pv. gardneri* ^b^			
Xg-28	Tomato	−	−
Xg-29	Tomato	−	−

^1^ Where *Xanthomonas* strains were isolated. ^a^ Previously *X. euvesicatoria* and ^b^ previously *X. gardneri*.

**Table 2:** Genomic features of *Xanthomonas* jumbo phages.

Feature	XaC1	XbC2
Genome size	348,967 bp	367,901 bp
% GC	35.1	37.5
ORFs	550	557
ORFs with homology	124	141
Hypothetical proteins	426	416
tRNAs ^1^	8	34
Lifestyle ^2^	Lytic	Lytic
Virulence factor ^3^	No	No
Antibiotic resistance genes ^4^	No	No

^1^ Determined with Aragorn V.1.2.41 and tRNA-SCAN-SE v.2.0. ^2^ Determined with PHACTS. ^3^ Determined with VFDB and Virulence Finder. ^4^ Determined with ResFinder.

## Data Availability

The original contributions presented in the study are included in the article/Supplementary Material, further inquiries can be directed to the corresponding author/s.

## Supplementary Materials

The following supporting information can be downloaded at: https://www.mdpi.com/article/10.3390/antibiotics13070651/s1, Table S1: Genome assembly statistics for *Xanthomonas* phages; Table S2: Annotation of XaC1 phage genome; Table S3: Annotation of XbC2 phage genome; Table S4: Identification of genes encoding tRNAs in XaC1 genome; Table S5: Identification of genes encoding tRNAs in XbC2 genome; Table S6: Calculation of codon frequency and codon usage of XaC1 and XbC2 phages; Table S7: Comparison of host usage percentage and the presence of phage-encoded tRNAs in XaC1; Table S8: Comparison of host usage percentage and the presence of phage-encoded tRNAs in XbC2; Table S9: Classification of identified ORFs of XaC1 phage by functional category; Table S10: Classification of identified ORFs of XbC2 phage by functional category; Table S11: Intergenomic similarity using VIRIDIC. Material S1: XaC1 Phage Term analysis; Material S2: XbC2 Phage Term analysis; Material S3: Restriction profile of XaC1 phage; Material S4: Restriction profile of XbC2 phage.
