# Supplementary material for: Isolation and Characterization of Two Novel Genera of Jumbo Bacteriophages Infecting Xanthomonas vesicatoria Isolated from Agricultural Regions in Mexico
Source: Antibiotics (Basel). 2024 Jul 15;13(7):651. doi: 10.3390/antibiotics13070651 (PMC11273794; doi:10.3390/antibiotics13070651)
Supplement: Supplementary file 1 [file antibiotics-13-00651-s001.zip › Material S3.pdf]

Name: XaC1

Conformation: linear

Enzymes: EcoRI, EcoRV, NdeI

Noncutters:

Name	Sequence	Site Length	Overhang	Frequency	Cut Positions
EcoRV	GATATC	6	blunt	27	5062, 5080, 18506, 18800, 19760, 20228, 25195, 26593, 30188, 46366, 70650, 106976, 164169, 167306, 169111, 214413, 227544, 237933, 248954, 276996, 283085, 307177, 314479, 332609, 335230, 346892, 348798
EcoRI	GAATTC	6	five_prime	140	34650, 35361, 35907, 37586, 41330, 45461, 45763, 46458, 49914, 51452, 52621, 53443, 53473, 53919, 61826, 62888, 67950, 69226, 71310, 73384, 74711, 74980, 75867, 76218, 77678, 79156, 80241, 81299, 81527, 82978, 83323, 92478, 106734, 107963, 108319, 108757, 114943, 117798, 117963, 118286, 119377, 119913, 122657, 123361, 125097, 125409, 125985, 127832, 129180, 133198, 134083, 134280, 135980, 138617, 149187, 149382, 151780, 161858, 166744, 176871, 178190, 179280, 179739, 180221, 187376, 187512, 188463, 189686, 190332, 190535, 191431, 193118, 193292, 195741, 197132, 203303, 208057, 208396, 209653, 209744, 211217, 217967, 225300, 226152, 230601, 232763, 235107, 240908, 241063, 243694, 244587, 244599, 244704, 246968, 250398, 250982, 255155, 255530, 255555, 255712, 256638, 261549, 262998, 264765, 269447, 269669, 270917, 272307, 274225, 279081, 281757, 284810, 285802, 288349, 289070, 289363, 293516, 295162, 303541, 303751, 303861, 304434, 307121, 307193, 311303, 318669, 320775, 322297, 325841, 326766, 326951, 327879, 330406, 331598, 332007, 333392, 336345, 338801, 339377, 340651
NdeI	CATATG	6	five_prime	162	1679, 6786, 18572, 19138, 21660, 24177, 27176, 28754, 28775, 30673, 30955, 33258, 33896, 38922, 39162, 42597, 42784, 44821, 48362, 49628, 50231, 52551, 53609, 55484, 56435, 59611, 60150, 61094, 61796, 63342, 64394, 69838, 70788, 71327, 72657, 72861, 73416, 80664, 81898, 85886, 87412, 89190, 89982, 93084, 95386, 97130, 99904, 100379, 103380, 106463, 106818, 109597, 110277, 110940, 111650, 114391, 114913, 115015, 115384, 118219, 118849, 119020, 120368, 123231, 125336, 128211, 128903, 129239, 130079, 132177, 136015, 137661, 139513, 139630, 146741, 153045, 153800, 156388, 163361, 163687, 164020, 168336, 168710, 171412, 178385, 179933, 180784, 182128, 191333, 192266, 195602, 195638, 195647, 198646, 199384, 201341, 202043, 203845, 208104, 208797, 208920, 209827, 211385, 213917, 214821, 225434, 228064, 229017,

Name	Sequence	Site Length	Overhang	Frequency	Cut Positions
					238536, 238707, 241887, 241985, 242126, 247002, 247094, 251146, 252235, 261800, 262541, 264073, 268314, 268386, 271832, 271922, 272600, 274286, 277446, 277812, 279945, 281749, 282272, 282437, 283520, 286009, 287685, 287712, 288519, 291680, 295198, 296007, 296543, 300117, 300837, 301821, 302821, 303623, 303931, 312777, 312837, 314107, 315087, 318423, 319511, 320067, 324287, 335065, 336443, 337480, 337570, 337810, 338289, 342982
