# Supplementary material for: Isolation and Characterization of Two Novel Genera of Jumbo Bacteriophages Infecting Xanthomonas vesicatoria Isolated from Agricultural Regions in Mexico
Source: Antibiotics (Basel). 2024 Jul 15;13(7):651. doi: 10.3390/antibiotics13070651 (PMC11273794; doi:10.3390/antibiotics13070651)
Supplement: Supplementary file 1 [file antibiotics-13-00651-s001.zip › Material S4.pdf]

Name: XbC2

Conformation: linear

Enzymes: EcoRI, EcoRV, NdeI

Noncutters:

Name	Sequence	Site Length	Overhang	Frequency	Cut Positions
EcoRV	GATATC	6	blunt	9	4388, 8809, 83399, 139105, 179294, 258149, 261739, 342285, 367722
NdeI	CATATG	6	five_prime	148	3254, 12730, 13565, 13964, 14970, 17645, 20533, 22941, 24718, 25727, 28474, 31002, 33568, 34770, 35327, 42158, 43533, 53416, 54324, 56051, 56441, 58205, 59459, 61268, 63841, 69452, 69537, 72279, 76958, 78086, 79165, 79172, 80615, 80771, 81242, 82148, 83207, 83555, 85282, 87487, 88077, 100475, 106637, 110534, 112497, 112706, 113791, 115874, 119191, 119332, 132048, 134852, 135341, 141510, 144998, 150537, 153936, 154191, 154326, 155493, 155907, 156492, 157339, 158136, 158250, 159474, 161617, 169033, 169090, 174262, 174337, 174429, 179160, 182293, 185592, 188417, 189526, 190758, 192650, 193078, 197057, 198284, 198984, 202475, 202577, 203169, 206900, 209144, 230225, 236119, 236651, 238376, 239891, 241128, 241317, 241674, 242073, 242424, 247634, 248509, 257750, 259419, 259551, 261649, 262229, 265074, 265330, 266163, 267251, 268891, 269110, 275402, 277939, 279767, 280447, 282639, 289375, 292997, 298069, 300973, 305815, 317350, 319828, 321152, 322755, 325071, 326310, 329993, 334993, 335722, 336232, 338053, 339981, 340089, 344430, 347115, 350554, 350979, 352989, 354294, 354660, 357638, 359456, 364960, 366190, 366257, 366704, 366902
EcoRI	GAATTC	6	five_prime	199	8593, 9912, 14527, 14957, 17685, 22107, 22613, 23043, 24064, 24399, 25772, 27381, 27675, 30973, 34883, 37306, 37933, 38993, 39705, 39849, 40388, 41307, 43508, 44933, 45197, 47135, 49388, 51281, 51834, 53366, 53648, 53847, 55390, 57194, 61861, 64618, 64856, 66517, 66553, 67177, 68539, 68802, 70841, 71830, 73334, 74553, 76022, 76596, 79748, 82183, 82488, 83998, 85224, 87240, 89210, 89528, 91473, 95842, 97146, 97870, 99309, 100426, 101292, 103356, 105267, 111953, 112588, 113412, 114050, 116471, 120273, 125126, 125282, 127555, 128266, 128485, 129295, 134063, 134476, 134606, 134765, 138290, 139999, 140933, 142010, 149022, 149034, 149370, 153493, 156522, 158694, 160066, 160291, 165356, 170414, 172079, 179514, 180440, 181615, 181753, 183473, 189266, 190446, 193353, 194371, 194413, 195418, 196944, 202008, 204994, 205776, 206575, 206589, 207439, 210171, 212308, 212811, 215595, 216399, 218954, 225356, 225520,

Name	Sequence	Site Length	Overhang	Frequency	Cut Positions
					225668, 226687, 228796, 228858, 230299, 230817, 232812, 233560, 237288, 237688, 238642, 242690, 243292, 246426, 247752, 247911, 248933, 251480, 253104, 257559, 257724, 258726, 260167, 261432, 262692, 263128, 263485, 265289, 266157, 267099, 269357, 269766, 270652, 270667, 271189, 271226, 273576, 273936, 274647, 275107, 278964, 283434, 284256, 286234, 286937, 289790, 292561, 293722, 295062, 295572, 296922, 300370, 301979, 308529, 309058, 309184, 312165, 314431, 316667, 316712, 324736, 328566, 328605, 331451, 336686, 337676, 339965, 340753, 342346, 343993, 344128, 345226, 348212, 352197, 353529, 356246, 363871
